# Hernia

**Published:** 1875-05

**Authors:** J. H. Pooley

**Affiliations:** Yonkers, N. Y.


ATLANTA

### Medical and Surgical Journal.

Vol. XIII.] MAY—1875. [No. 2.

### Original Communications.

### HERNIA.

By J. H. Pooley, M.D., of Yonkers, N. Y.

Hernia is one of the *great* subjects of surgery. In every text-book, both of surgery and anatomy, it occupies a large and prominent place, while many separate treatises are devoted to its consideration, among which ought to be mentioned, as not only admirable works on the subject, but as models for all surgical monographs, the great works of Lawrence, Sir Astley Cooper, and Scarpa, the latter accessible to the English reader in the translation of Wishart.

Besides being so abundantly treated of in books, the subject of hernia, particularly the demonstration of its anatomy, is one to which several lectures of every surgical and anatomical course in our medical colleges are devoted, to the weariness not only, but often to the life-long confusion, of the poor student.

The oldest medical works contain very complete accounts of this disorder, and scarcely a week goes by without one or more cases being reported in the journals. The importance thus attached to the subject of hernia by the surgical mind of all ages arises mainly from three circumstances:

1st. Its frequency. It is an extremely common disease, being variously estimated to prevail in from one-eighth to one-sixteenth of the human family; and if the first of these estimates is too large, as it very probably is, the latter is quite as probably too small. Without citing any authorities, among whom such a wide diversity exists, I will simply remark that the accurate determination of such a question must necessarily be extremely difficult, if not impossible, as many of those thus afflicted take the greatest pains to conceal their infirmity, and all statistics on such a question, drawn from hospitals, armies, or similar sources, are apt to rate the proportion too high, representing, as they do, but one class of society, and that too in which the disease is confessedly most common. The great difference found to exist among those authorities who have pretended to give figures on this question shows plainly enough that none are to be implicitly relied on, and perhaps the subject admits of no more definite statement than that hernia is one of the most frequent of surgical affections. How often are we surprised when some emergency makes us acquainted with the existence of hernia in a person whom we have known for years without in the least suspecting it.

2d. Its serious risks. Hernia, although at ordinary times, and when quiescent, but one of the minor ills of life, is liable at any moment to become one of the most painful, dangerous and urgent of morbid conditions; threatening a speedy period to a life which but a few hours before seemed almost out of the reach of death or danger, and demanding upon the part of the medical attendant the utmost promptitude and coolness of action. No accident to which so many people are liable is so terrible, or so frequently mortal, as strangulation of a hernial tumor.

3d. Its manifold and unexpected complications. The complete history of hernia will never be written, for no two cases are in all points exactly alike, and something new is always "turning up." Complications are constantly arising which tax to-day, as they have done for hundreds of years, all the skill and resources of the surgeon, who, though he may know nothing certain of the conditions before him, has no way of attaining that certainty but by acting as if he were already acquainted with every detail. Wonderful cases of hernia have been constantly recurring in medical literature, from the day when Pott found the ovaries in a hernial sac, to the probably unique case reported in the *New York Medical Journal* for January, 1873, where the intestine was strangulated by the spermatic cord.

These three things, then, its frequency, its danger, and its complications, will always make hernia, as they always have made it, one of the most interesting and important subjects in surgery. I have not, as may well be supposed, chosen the subject of hernia for a paper because I hope to exhaust it, for that would be all that could be expected in the most pretentious volume; nor because I have anything specially new to offer, but because it has occupied much of my reading and meditation, and because I have seen abundant reason to believe that many of the profession either entertain erroneous notions on the subject, or display in dangerous emergencies a culpable timidity and delay. The nature of this paper forbids any effort at systematic arrangement, or even thorough elaboration of the points on which it touches. I intend only to take up a few points as they occur to my mind, and discuss them very briefly, guided by the time-honored advice of the Irishman to a friend in difficulty, "whenever you see a head, hit it."

And first, I wish to make a few remarks on some points connected with hernia in children. It is important, of course, that every case of hernia, whether in an adult or child, should be supported by a proper truss; but this is of double importance in children, from the fact that what in the adult is a mere paliative, may in their case produce a radical and permanent cure. A well-fitting, carefully-applied, constantly-worn truss will, I am persuaded, very often produce a perfect cure. But so desirable a result is not to be obtained without some considerable amount of care and trouble. In the first place, the truss should be adapted as perfectly as possible to the requirements of each particular case, and this is sometimes by no means an easy task; it is generally useless, it may be a great deal worse than useless, to be satisfied with simply issuing the mandate, "get a truss and let the child wear it."

A badly-fitting truss can seldom be worn long enough to do any good, and is utterly useless for the purpose of cure, even if persevered in. Let the surgeon, if possible, select the instrument himself, but as this may not always be convenient, let the patients, in applying to the instrument maker, arrange for the exchange of the truss as often as may be necessary, until the surgeon passes a full verdict of approval. And in this matter let us beware not to decide too hastily; more than once have I seen a truss pronounced perfect, and seeming to fit perfectly when first applied, in a day or two develope faults that rendered a change imperative; indeed, I think it might almost be laid down as a rule, that not until after one or two days' wear can a safe decision be arrived at. And even after a proper instrument has been selected, and the parents or nurse have been shown and instructed how to apply it, the surgeon should not at once cease personal oversight of the case, but should, for a few weeks at least, carefully examine it from time to time to see that it is working properly.

A truss for a child ought always to be selected and worn with a view to a radical cure, for though we may sometimes fail in attaining such a desirable result, we shall succeed in a sufficient number of cases to make it worth while to try for it in all. I can recall at this moment at least four little patients who have been thoroughly cured of scrotal hernia by carefully wearing a truss for from one to two years, and I by no means think this latter time the latest period at which such a result may be hoped for. To attain so desirable an end, the truss must not only support the hernial protrusion and retain it within the cavity of the abdomen, but it must also produce enough pressure upon the internal abdominal ring and first part of the inguinal canal to occlude, or at least to diminish, these apertures. Pressure sufficient to do this will, of course, cause the child some pain and uneasiness, particularly at first, but this need not be severe, indeed it will not be borne if it is; to regulate it will require some judgment, which no rules can supplant or materially assist, and which must result from close attention to each case, which will enable the practitioner to conduct this apparently simple, but really important matter in the best way.

The truss ought to be worn unremittingly, night and day, and for an entire year at the very least. To carry this out in its full rigor will, I am well aware, generally be impossible just at first, and, on the whole, had perhaps better not be attempted; for the first month perhaps it may be taken off at night, and reapplied in the morning, and great comfort may be given the little patient by bathing all the parts upon which pressure is made with equal parts of alcohol and water, both after taking it off and before putting it on again.

How soon may a truss be applied for infantile hernia? I answer, as soon as it is discovered, and if congenital, within the second month. This answer, however, admits of one exception, and that is where there is an undescended testicle on the same side as the hernia; in such a case it might be well to wait for six months or so, but not much longer, for if the testicle has not descended by that time, the probabilities of its ever doing so are very small.

If a truss is applied during the early months of infantile life, it will need to be changed from time to time to meet the demands of growth; and even if it were not necessary on this account, it would be on others, for the constant soiling and wetting to which it is liable will soon wear out and destroy the leather and softer parts of it at least, and I have known the steel of the spring to become so rusted and weakened as to snap right across on the application of a very trifling force. Every time the truss is changed, one with a stronger spring should be selected.

Let us bear in mind that it is not the application of a severe pressure, but the continuance of a more moderate degree that we are to rely upon, and that there is no special secret, either of form or material, that can make the instrument of one maker universally superior to all others, but that each may have something to commend it for some particular case, and thus we shall have a wide field to choose from, and can rarely fail, by perseverance, to find what we need.

Proprietors or patentees of different trusses may have their hobbies, but the practical surgeon should have none. I had recently a very severe case of double scrotal hernia in an infant, the tumor altogether being larger than a man's fist, forming a projection, in the midst of which the little penis was almost buried out of sight, and over which the urine dribbled, producing a good deal of superficial excoriation, and it was growing bigger all the time.

A number of trusses were tried, at the expense of a good deal of time and money, but to no purpose; and one maker to whom we applied said that it was the only case in which his truss had failed, and the rupture was so bad that no truss could control it. Nevertheless, I was not to be daunted, and turned truss-maker myself, theoretically at least, and finally succeeded in having one made for the case which kept it up perfectly, and has done so in spite of a severe attack of whooping-cough, and it now seems in progress of cure. Let us never despair; there is almost always a way, and we can almost always find it if we try hard enough, and long enough

In the scrotal hernia of children, we sometimes find a small quantity of fluid at the bottom of the sac, constituting a variety of hydrocele. Especially is this apt to be the case in congenital hernia. It rarely ever requires any separate attention, but generally disappears in a short time when the hernia is retained in its place. If in any exceptional case it should be deemed advisable to puncture it, the greatest care would be necessary to make sure that the intestinal contents were first replaced, and a truss applied, and in no case would irritating injections be admissible.

I have met with a source of uncertainty in some cases of hernia in children, which only needs to be known to be escaped. I refer to the fact that owing to the looseness of their tissues there will often appear to be something left in the sac after the hernial contents have been properly reduced. A knowledge of this source of perplexity, and a moment's attention, will suffice to prevent any mistake. It is but rarely that any considerable difficulty is experienced in reducing the hernia of children, but should there be, I have found the plan of turning the little patient literally upside down, by suspending him by the heels, a most efficacious one. The first time I tried it was in a case in which I had been sent for to operate on a child for strangulated hernia. Before proceeding to operate, I determined to make one final effort at reduction, and directed the father to stand upon a chair, and taking his child by the heels, let him hang head downwards. In this position the hernia, which had previously resisted many efforts at reduction, went back almost of itself, without the assistance of the taxis. This plan, which cannot be fully carried out in an adult, should always be tried, if necessary, in children; nor would I say that the taxis had finally failed until it had been done. In adults an approximation may be made to it by lowering the head and shoulders and elevating the pelvis as much as possible, though it has been advised to attempt the taxis with the patient in the upright position. I have no experience with this method, but there are plenty of cases where one is glad enough to try anything, and this will not be the first instance in which diametrically opposite means have been successful in attaining the same result. One important point in the position of a patient to facilitate taxis, which is often overlooked, is that the thigh on the side of the hernia should not only be flexed, but also strongly adducted, or rotated inwards.

There is a form of hernia which is seldom seen in the adult, but is quite common in children, that is the umbilical, which frequently exists at birth, or more commonly shows itself soon afterwards; it generally cures itself, either with or without the assistance of the physician. The only treatment I have ever used or found necessary has been to place a compress, a penny piece, for instance, wrapped in muslin, over the opening, and retain it by cross strips of adhesive plaster, directing the mother to pin the customary bandage a little tighter than usual, and keep her child from crying as much as possible. The latter direction, as also the prevention of constipation and its accompanying straining, is of importance in all cases of hernia.

A case of this kind was brought to me a few days ago in which some wise neighbor woman had offered to cure it by a puncture, but the parents, though they had great confidence in her knowledge, preferred, as they said, to have a *regular surgeon* perform the operation; so they came to me, and the little baby escaped an artificial anus, perhaps, if not something worse. Such cases have not always fared so well, for they have sometimes been opened, and death or artificial anus produced, and I have seen lately a notice in one of the journals that, in a case of congenital umbilical hernia the same misfortune has been caused by tying the cord too near the body of the child. When umbilical hernia occurs in the adult, it is generally in women, and is caused by the thinning and spreading out of the abdominal walls in pregnancy, and the expulsive efforts of parturition, and is often extremely annoying and difficult to deal with.

I have said already that when a hernia becomes strangulated we have a comparatively unimportant disease suddenly changed to one of the utmost danger and gravity, and one too in which the surgeon must act with promptitude and decision, if he would magnify the efficacy of his art, instead of having to deplore its uselessness. I have known, in a not very extensive experience, several cases of strangulated hernia terminating fatally by delay, and facts such as these explain why I speak so emphatically and with such repetition on so plain a matter.

I shall not stop to describe the symptoms of strangulation, taking it for granted that we all know them well enough, but simply say that when once this condition is ascertained to exist, or even to be imminent, the surgeon should give himself no peace nor rest until it is overcome, either by taxis, or, failing this after a fair trial under anæsthesia, by operation. We must not, now-a-days, discuss the influence of bleeding, antimony, tobacco enemata, etc., for although useful to our forefathers, they are, as a general rule, unnecessary and superfluous in the practice of to-day. We have anæsthetics, and this simple fact gives us such an unspeakable advantage in the treatment of hernia, as well as many other conditions, that there is no longer any excuse for torturing our patients with delays and debilitants that were once all but unavoidable. In the use of anæsthetics we have not only the most perfect means of relaxation, with less of danger and inconvenience, than almost any other, but moreover as a means of abolishing pain, it delivers us from that most fatal of temptations, delay, after the taxis has been tried and failed.

I am well aware that our art admits of but few, if any, *absolute* rules of conduct, but that those considered most undeviating often demand variation according to the judgment of the practitioner in particular cases; nevertheless, the following rule for the guidance of the surgeon, when called to a case of strangulated hernia, will probably demand as few exceptions as any in surgery. When called to such a case make an immediate effort at reduction, with the aid of an anæsthetic if necessary and practicable. This attempt may be continued for half an hour or more if there be no tenderness or other evidence of local inflammation; otherwise it must be discontinued sooner, according to circumstances. Let the taxis be systematic, gentle and persevering; force is never appropriate, for even if it succeeds it is dangerous. If after a fair trial in this way you fail, then send for assistance, and appoint a consultation at the earliest possible moment, and come to it prepared to operate. Place your patient fully under the influence of ether, or chloroform if you like it better; try the taxis once more, and if it fails operate at once.

By adopting such a course as this you will very often come away without having used your instruments; for in the large majority of cases taxis under ether succeeds; but you will always come away conscious that you have done all you could to place your patient in a position of safety and preserve his life—all at least that a good conscience and good surgery demand, and they certainly demand no less of you. Though you should always be prepared to operate in such cases, never regard an operation for strangulated hernia as trifling or unimportant. It is no trifle, but a very grave and serious operation, and one, too, having a very high rate of mortality—not less than twenty-five per cent.; but it is far, *far* less dangerous than delay. Indeed, it is injudicious delay that has done much to give it its unenviable mortality; and, like many other of the life-saving operations of surgery, its true value will never be appreciated until it ceases to be regarded as the last resort—the only step short of sheer despair—done on moribund patients, or simply to quiet the demands of a half-enlightened conscience.

Having thus spoken briefly of the relative position of taxis and the knife, let us look at each more in detail for a moment.

We have said already that taxis, with the patient thoroughly anæsthetized, succeeds in the large majority of cases; we may go further, and say that if properly performed, and undertaken in time, in cases of inguinal hernia, it almost always does succeed. The cases in which it oftenest fails are those of femoral or crural rupture, the reasons for which are, the small size of the aperture through which it has escaped, the depth and situation of the parts, which prevent the proper manipulation of the sac, and render it liable to become doubled or folded up against the sharp edge of Gimbernat's ligament, where the stricture is generally situated, and which, interrupting the circulation more thoroughly than the comparatively rounded edge of the other outlets, causes swelling to occur more rapidly and more severely. We need not stop to give any directions for the performance of the taxis. There is plenty of that in every text-book on surgery, and I have nothing new to offer. Only do it reflectively, thinking carefully all the while, even with your finger ends, and bearing your anatomy carefully in mind. I once watched a doctor for a considerable time sweating and puffing in vain over the reduction of a hernia, which after all proved quite easy in other hands. The only difficulty was he was pushing in the wrong direction. Above all do not resort to what may be called brute force. Please remember that these are living, sensitive tissues you are pushing, and kneading, and twisting, and squeezing; nothing but a few thin layers lie between your ruthless fingers and delicate, vascular, and it may be already partially inflamed, intestine.

If, after the use of force and violence, you are obliged to operate, you more than double the risk of fatal traumatic peritonitis. The celebrated Desault was so fully convinced of the great danger of immoderate efforts in applying taxis that he condemns it in almost every instance. In his opinion the bruising and other injuries inflicted on the bowel by the surgeon in such attempts may render the state of the patient as critical after the reduction, *if accomplished*, as it was before.

Desault has seen many cases that tend to show a great difference in the mortality after operating, in favor of those operations which have been performed on patients who have not previously been subjected to the taxis. "You may always hope for success," he says, "in a hernia which has not been touched before operating." Now, with all due deference to so celebrated a surgeon as Desault, I think that to recommend the operation without any previous attempt at reduction, is going to an unreasonable and unjustifiable extreme; and yet I am almost sorry to be obliged to enter this *caveat*, so impressed am I with the supreme danger of delay, and the continuous and prolonged intermeddling which it almost invariably involves. And moreover, as already remarked, the triumph of obstinate force is often but a fallacious one, even when it succeeds in effecting reduction. I have seen this strikingly exemplified at least once in a patient who, after such a triumph of muscular energy, escaped death by the very "skin of his teeth." The use of ether spray as an adjuvant to the taxis, has been repeatedly used and advised within the last few years; its actual value remains to be tested by further clinical experience, which is the only unfailing touch-stone by which every new expedient must stand or fall.

Having said that in undertaking the taxis we should carefully remember our anatomy, I now remark that when we come to operate the more completely we leave it out of mind the better. Strange doctrine! but nevertheless I believe it to be true. I scarcely think it worth while to guard myself against misapprehension by saying that I by no means underrate the importance of anatomy, for no one can have a higher estimate of its paramount importance than I have; but I cannot help thinking that a man who begins an operation for strangulated hernia expecting to find the layers and faciæ he has read about in books, or even seen in the dissecting-room, is inflicting the most useless torture on himself, and doing his patient no earthly good.

Just think of it; what is it that you have got to do? Not to demonstrate any relation of parts, either real or fancied, but simply and only to cut down to the seat of stricture and relieve it, be it where it may. There would seem to be but little fear of rashness, for you know well enough that the intestine, no matter how many layers ingenuity may divide the superjacent tissues into, is practically very near the knife, and to cut into it would be such a blunder as no amount of anatomical knowledge could avert. And is it to be supposed that any operator knows or cares, as he divides layer after layer on his dissector, what their names may be? The only excuse I can imagine for such useless trifling would be the positive recognition of the hernial sac itself, by having cut through the number of layers which must necessarily precede it; but, as everyone is aware, no such rule is available, and if it were it would be of little use, except to one who could not recognize the sac when he saw it, and he could hardly be made a safe operator by any rules. No; care, deliberation, and good common sense are the best safeguards; and even with regard to arteries, as being on the inside of this sort of hernia, and on the outside of that, they are niceties that we are least likely to feel sure about just when we need that certainty most, and such rules are by no means certain and reliable; the artery is not always where it ought to be. Let the finger precede the knife in dividing the stricture, carefully feeling for the pulsation of an artery, or anything else of which the tactile sense can inform us, and, unless warned of some danger, cut directly upward, where danger is least likely to be met. With regard to the division or opening of the sac, that is, of the peritoneum, this should always, if possible, be avoided. Notwithstanding the great stress laid by the advocates of certain operations upon the impunity with which the peritoneum may sometimes be wounded, let us not be led astray. Fear not to divide it when necessary, but avoid it when possible, for, as already stated, the mortality of operations for strangulated hernia is high, and this mortality is chiefly from traumatic peritonitis.

The tendency of many patients to conceal the existence of a hernia, and the fact that it may even exist without their knowledge, make it important, under certain circumstances, to be on the lookout for it. In every case of colic, especially if there be vomiting, it would be well to determine whether there is a hernia or not. Do not be led to neglect this necessary precaution by the fact of the bowels having been moved; so far from this deciding against the presence of hernia, the fact is that a fæcal discharge, and a very free one, quite often occurs at the moment of strangulation. Therefore, always examine the usual situation, which can easily be done while ascertaining the condition of the abdomen as to tenderness, distension, etc. It will not do to trust to questions merely, for the patient, from motives of delicacy, from ignorance, or because he takes a different view of the case, may put you off.

Many patients have died of so-called colic, and a strangulated hernia been discovered only after death. If such an occurrence should happen to any of us, it would make us very uncomfortable, and if by such a simple precaution we should save one patient that would otherwise have died, that would be reward enough for having practised it all our lives.

I am aware that this paper is both imperfect and commonplace enough, but it is well sometimes to go over well-known ground again and again, and trifles acquire great importance when they pertain to a matter so serious as the one we have been considering.

